# A novel and stable "two-hit" acute lung injury model induced by oleic acid in piglets

**DOI:** 10.1186/1751-0147-51-17

**Published:** 2009-03-30

**Authors:** Xiaofeng Li, Yinglong Liu, Qiang Wang, Yaobin Zhu, Xiaodong Lv, Jinping Liu

**Affiliations:** 1 Peking Union Medical College and Chinese Academy of Medical Sciences, Pediatric Cardiac Surgery Center, Cardiovascular Institute and Fuwai Hospital, Beijing, PR China; 2 Peking Union Medical College and Chinese Academy of Medical Sciences, Department of Cardiopulmonary Bypass, Cardiovascular Institute and Fuwai Hospital, Beijing, PR China

## Abstract

**Background:**

Children are susceptible to pulmonary injury, and acute lung injury (ALI) often results in a high mortality and financial cost in pediatric patients. Evidence has showed that oleic acid (OA) plays an important role in ALI. Therefore, it has special significance to study ALI in pediatric patients by using OA-induced animal models. Unfortunately, the animal model hs a high mortality due to hemodynamic instability. The aim of this study was to establish a novel hemodynamically stable OA-induced ALI model in piglets with two hits.

**Methods:**

18 Chinese mini-piglets were randomized into three groups: group C (received saline-ethanol solution), group T (received OA-ethanol solution in routine administration manner) and group H (received OA-ethanol solution in two-hit manner). Hemodynamic and pulmonary function data were measured. Histopathological assessments were performed.

**Results:**

Two piglets in group T died of radical decline of systemic blood pressure. Group T showed more drastic hemodynamic changes than group H especially during the period of 5 to 30 minutes after OA administration. Both Group T and group H all produced severe lung injury, while group C had no significant pathologic changes. OA-induced hypotension might be caused by pulmonary hypertension rather than comprised left ventricular function.

**Conclusion:**

OA leads to severe pulmonary hypertension which results in hemodynamic fluctuation in OA-induced ALI model. It is the first report on hemodynamic stable ALI animal model in piglets using two-hit method. The two-hit ALI animal model fulfils the ALI criteria and has the following characteristics: hemodynamic stability, stable damage to gas exchange and comparability with pediatric patients in body weight and corresponding age. The two-hit ALI animal model can be used to study the basic mechanism and the therapeutic strategies for pediatric ALI.

## Background

Because of pediatric patients' susceptibility to lung injury, acute lung injury in children often results in a high mortality [1]. Therefore, it is crucial to research its mechanism and the therapeutic strategies for those patients. In addition, the blood level of OA is significantly elevated in patients with acute respiratory distress syndrome (ARDS) [2,3], and the proportion of OA incorporated into surfactant phospholipids also increases [4,5]. These evidences suggest that OA may play an important role in ARDS. In order to have a thorough insight into the syndrome, researchers often employ OA-induced ALI animal model to study the role of OA in the pathological process of ARDS [6]. Unfortunately, the animal model is associated with a hemodynamic instability and a high mortality up to 30% [7-9], and, to our knowledge, there is no report of this kind of animal model in piglets whose body weights are comparable with infant patients'.

The present study was aimed to establish a stable "two-hit" ALI animal model induced by OA in piglets, f which the body weight and corresponding age were comparable with those of pediatric patients.

## Methods

### Animal preparation

The study protocol was approved by the Laboratory Animal Ethics Committee of the Chinese Academy of Medical Sciences and Peking Union Medical College. Eighteen experimental Chinese mini-piglets (7.84 ± 0.33 kg) were purchased from the National Laboratory Animal Center of China and were randomized into three groups: 1) control group (C); 2) traditional oleic acid administration group (T); 3) two-hit group (H). There was no difference in body weights among groups.

### Reagents

Oleic acid (cis-9-octadecenoic acid, OA) was purchased from Sigma (Sigma Chemicals, St. Louis, MO, USA) and was diluted in 96% ethanol (1:1 by volume).

### Surgical procedures and ventilatory conditions

All the piglets were anesthetized with pentobarbital sodium (30 mg/kg). Anesthesia was maintained by continuous infusion of pentobarbital sodium (8.5 mg/kg/h). Animals were placed in a supine position on an operating table and were intubated and ventilated (900c servo-ventilator, Siemens Elema, Solna, Sweden) with a volume-controlled ventilation mode, an inspired O_2 _fraction (FiO_2_) of 0.8, a respiratory rate of 20 breaths per min and a tidal volume (Vt) of 10–20 ml/kg to maintain arterial partial pressure of CO_2 _(PaCO_2_) between 35 to 40 mmHg before oleic acid administration. A positive end-expiratory pressure (PEEP) of 5 cmH_2_O was adopted when the ALI criterion was achieved. A double-lumen catheter was placed into the abdominal aorta via the right femoral artery for intermittently sampling of blood and continuously recording of systemic arterial pressure (Psa). Median sternotomy was performed, and the aorta was separated from the main pulmonary artery. A 16- to 20-mm nonconstricting ultrasonic flow probe (T101; Transonic Systems, Ithaca, NY) was positioned around the ascending aorta root to monitor the instantaneous cardiac output (CO). Three catheters were inserted for pressure monitoring and sampling: (1) a triple-lumen was inserted into the right atrial appendage for central venous pressure (CVP) monitoring, blood sampling and oleic acid injecting; (2) a double-lumen catheter was inserted into the main pulmonary artery root; (3) another double-lumen catheter was inserted into the left atrial appendage. All catheters were intermittently flushed with normal saline containing a low dose of heparin (10 IU/ml infusion fluid) to avoid clotting in the catheters.

### Experiment protocols

**Figure 1:**
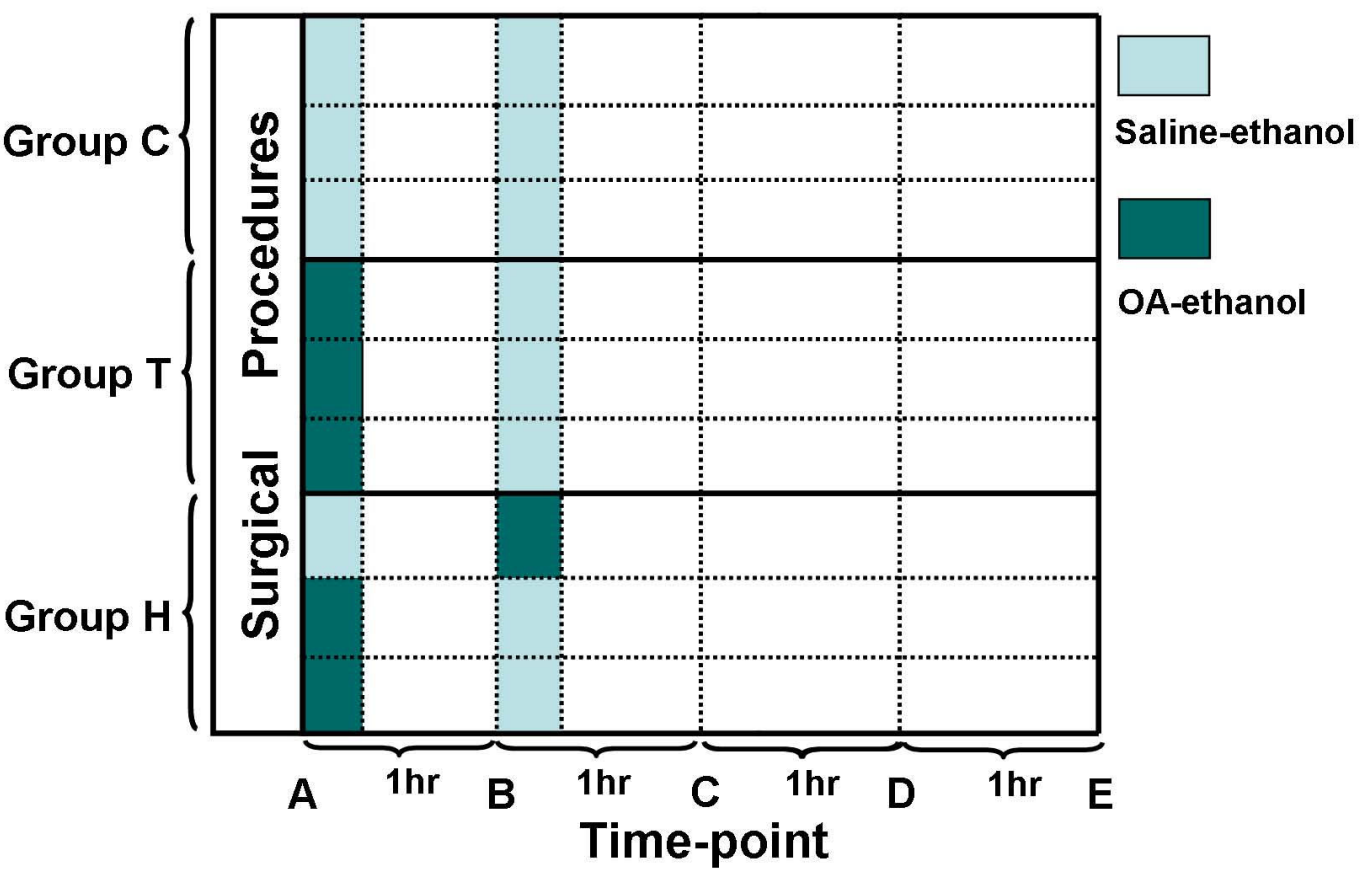
**The regimen of oleic acid administration and time-line of the experiment protocol**. Time-point A, 30 min after surgical procedure; time-point B, C, D and E, 1 hr, 2 hrs, 3 hrs and 4 hrs after time-point A. Group C received 0.1 ml/kg saline-ethanol solution at time-point A and B respectively; Group T received 0.1 ml/kg OA-ethanol solution at time-point A and 0.1 ml/kg saline-ethanol solution at time-point B; Group H received 0.07 ml/kg OA-ethanol solution followed by 0.03 ml/kg saline-ethanol solution at time point-A and 0.03 ml/kg OA-ethanol solution followed by 0.07 ml/kg saline-ethanol solution at time-point B.

The criterion for acute lung injury was defined as PaO_2_/FiO_2 _ratio <200 mmHg [10]. The stable ALI model was established if PaO_2_/FiO_2 _ratio did not elevated after 90-min stabilization. The timeline of the protocol was defined as follows: baseline data were acquired after 30-min stabilization from surgical procedures and the time point was defined as time-point "A" according to the timeline of group T. 1 hr, 2 hrs, 3 hrs and 4 hrs after time-point A was defined as time-point B, C, D and E (Figure 1) respectively. Group C received 0.1 ml/kg of saline-ethanol solution (1:1 by volume) at time-point A and B. Group T received 0.1 ml/kg OA-ethanol solution (1:1 by volume) at time-point A according to the traditional method [11] and 0.1 ml/kg saline-ethanol at time-point B. Injection of 0.1 ml OA within one second was given at an interval of 90 s. Group H received 0.07 ml/kg of OA-ethanol solution, about two thirds of the total dosage, at the time-point A serving as the first hit, and the other one third of the dosage was injected slowly in the second stage serving as the second hit at the time-point B. If a preceding injection caused a significant decline of Psa, the injection was slowed down. Normal saline was infused at a rate of 10 ml/kg/h throughout the experiment. In order to evaluate the stability and the true hemodynamic change trends, dopamine was not administrated. After finishing the protocol, all the animals were sacrificed under deep anaesthesia with a bolus injection of thiopental followed by 40 ml of potassium chloride i.v., and the lungs were harvested *en bloc*. Specimens from the inferior lobe portions of each lung were harvested and inflated with 10% formalin before processing for routine light microscopy. Tissue blocks (2 mm^3^) were inflation-fixed with 4% solution of glutaraldehyde for electron microscopy.

Ventilation parameters, such as peak airway pressure (Paw), PEEP and frequency, were recorded. A series of blood samples (0.6 ml) were taken for immediate blood gas analysis. Pulmonary function indexes were calculated by the following formulas as previously described[12]: PaO_2_/FiO_2 _ratio (P/F):

*P*/*F *= *PaO*_2_/*FiO*_2_

Alveolo-arterial Oxygen tension Difference (A-aDO2):



(Patm is the pressure of atmosphere)

### Hemodynamic measurements

Systemic arterial pressure (Psa), pulmonary arterial pressure (PAP), left atrial pressure (Pla) and central venous pressure (CVP) were continuously measured. Cardiac output (CO) was measured with transonic ultrasonic flow probes. A standard electrocardiogram was recorded.

### Histopathological assessment

Lung tissues were sectioned (4 μm) and stained with hematoxylin-eosin to quantify lung injury. Ten randomly selected fields per slide were read at ×400 magnification. Scoring was performed according to the methods reported previously [13,14]. All the lung tissue blocks also underwent transmission electron micrograph.

### Wet weight to dry weight ratio measurements

Lung tissue blocks were also obtained from right lungs for wet- to dry-weight ratio (W/D) measurement to evaluate the severity of pulmonary edema [15].

### Statistical analysis

All data were presented as mean ± SD. Statistical analyses among more than three groups were performed using one-way ANOVA. When significance was achieved, it was followed by post hoc analysis. Student's *t*-test for paired samples was also used. Statistical analysis was performed using SPSS (SPSS 13.0 for windows, SPSS, Chicago, IL, USA). We regarded difference with a *p*-value < 0.05 as statistically significant.

### Results

The main findings of this study, under the conditions of the present study, were as follows:

Two animals in group T died of extremely rapid decline of Psa shortly after OA administration, and only 4 passed through the protocol in group T. There was no death in group H before the end of the protocol. Mean Psa (MPsa) of the first death dropped from 98 to 43 mmHg 5 minutes after OA administration, then cardiac arrest happened. The other also died of hypotension 15 minutes after OA injection.

### Hemodynamic Effect of OA

**Figure 2:**
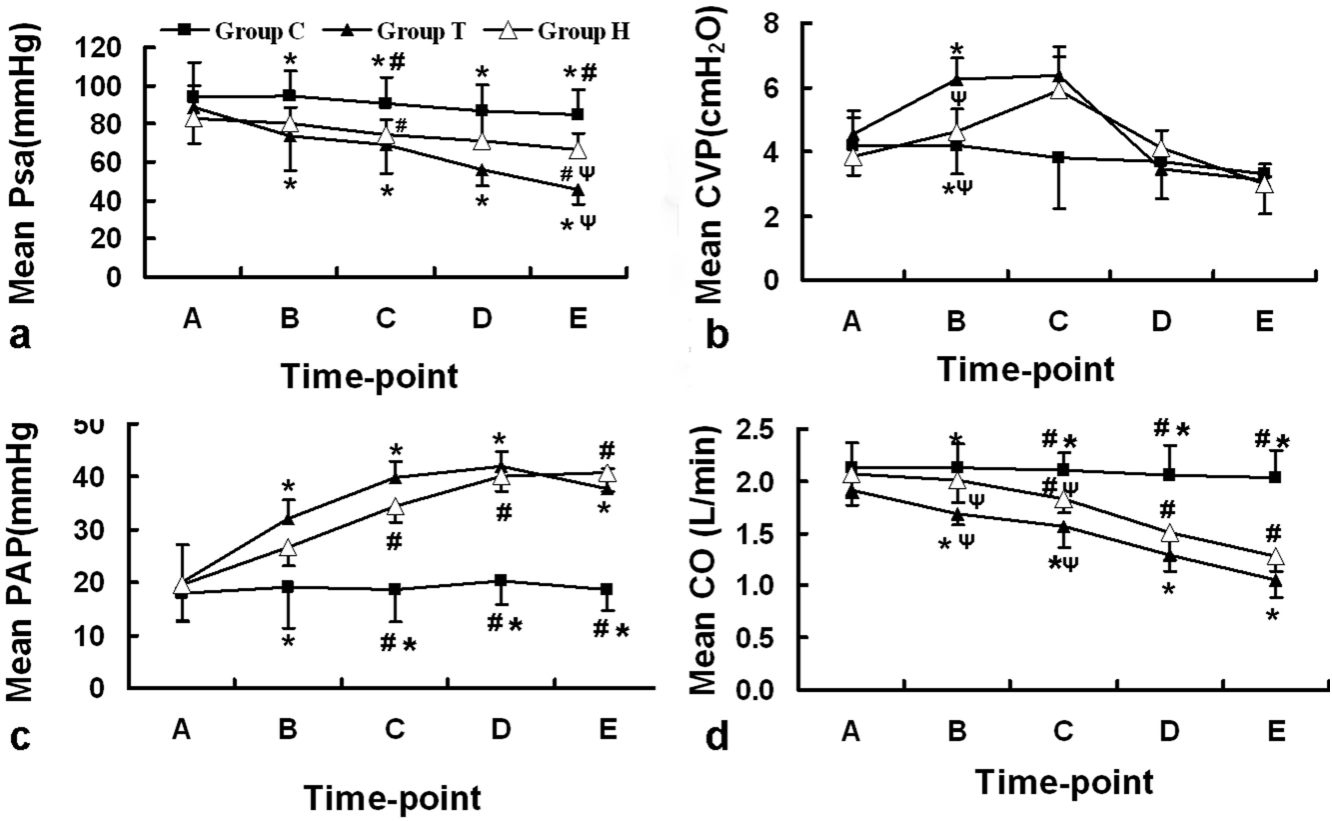
**The change trend of MPsa, MCVP, MPAP and MCO**. a, the change of mean Psa; b, the change of mean CVP; c, the change of mean PAP; d, the change of mean CO. * *p *< 0.05, group C comparing with group T; # *p *< 0.05, group C comparing with group H; Ψ*p *< 0.05, group H comparing with group T.

Mean Psa, CVP, PAP and CO were shown in Figure 2. All these indexes had no marked difference at the baseline. MPsa of group C was stable and showed no difference between every time points. MPsa in group T began to sharply decrease shortly after OA administration and declined to 45.9 ± 8.3 mmHg at the end of the experiment (*p *= 0.004 comparing with baseline). From the time-point of OA administration to the end of the experiment, the decline of MPsa (ΔMPsa) in group T was 42.6 ± 15.5 mmHg, which was significantly more than that of group C (9.4 ± 19.6 mmHg, *p *= 0.01) and group H (16.4 ± 15.4 mmHg, *p *= 0.034); however there was no significant difference in theΔMPsa between group C and group H (*p *= 0.491).

The authors found that the MPsa began to drastically decline 5 minutes after OA administration in group T, but that of two-hit animal models began to moderately decline 30 minutes after OA injection and showed a stable decline trend as group C. To evaluate the effect of OA on cardiac function, mean Pla (MPla) was monitored. MPla did not differ between groups (*p *> 0.05 respectively) and was not higher than 10 mmHg at every time points. Mean CVP (MCVP) of both group H and group T increased shortly after OA injury, but this trend didn't keep immutable throughout the experiment. In the last 2 hrs, MCVP of both group H and group T showed a decline trend. Mean PAP (MPAP) of group H and group T significantly increased from the baseline of 19.61 ± 5.04 mmHg to 26.55 ± 3.5 mmHg (*p *= 0.02) and from 20.11 ± 7.00 mmHg to 32.11 ± 3.39 mmHg (*p *= 0.033) in the first hr. MPAP of group T increase drastically between time-point A and time-point B in which period two deaths occurred, but it slightly declined in the last hr. On the contrary, MPAP of group H had a moderate increase trend. The CO of group C did not show significant changes, but CO of both group H and of group T showed a decrease trend. CO of group T in the first hr showed a drastic decrease trend from 1.92 ± 0.15 L/min to 1.68 ± 0.11 L/min (*p *= 0.042). The decrease of CO of group T in the first hr (ΔCO) was significantly more than that of group C and group H (*p *= 0.005 and *p *< 0.001 respectively), in which period the two deaths of animals happened. No difference inΔCO was found between group C and group H (*p *= 0.159).

At the end of the experiment, the CO of group T remained significantly lower than that of group C (*p *< 0.001). Although no difference was found between group H and group t (*p *= 0.110), the CO of group H seems slightly higher than that of group T

### Pulmonary function and blood gas results

**Figure 3:**
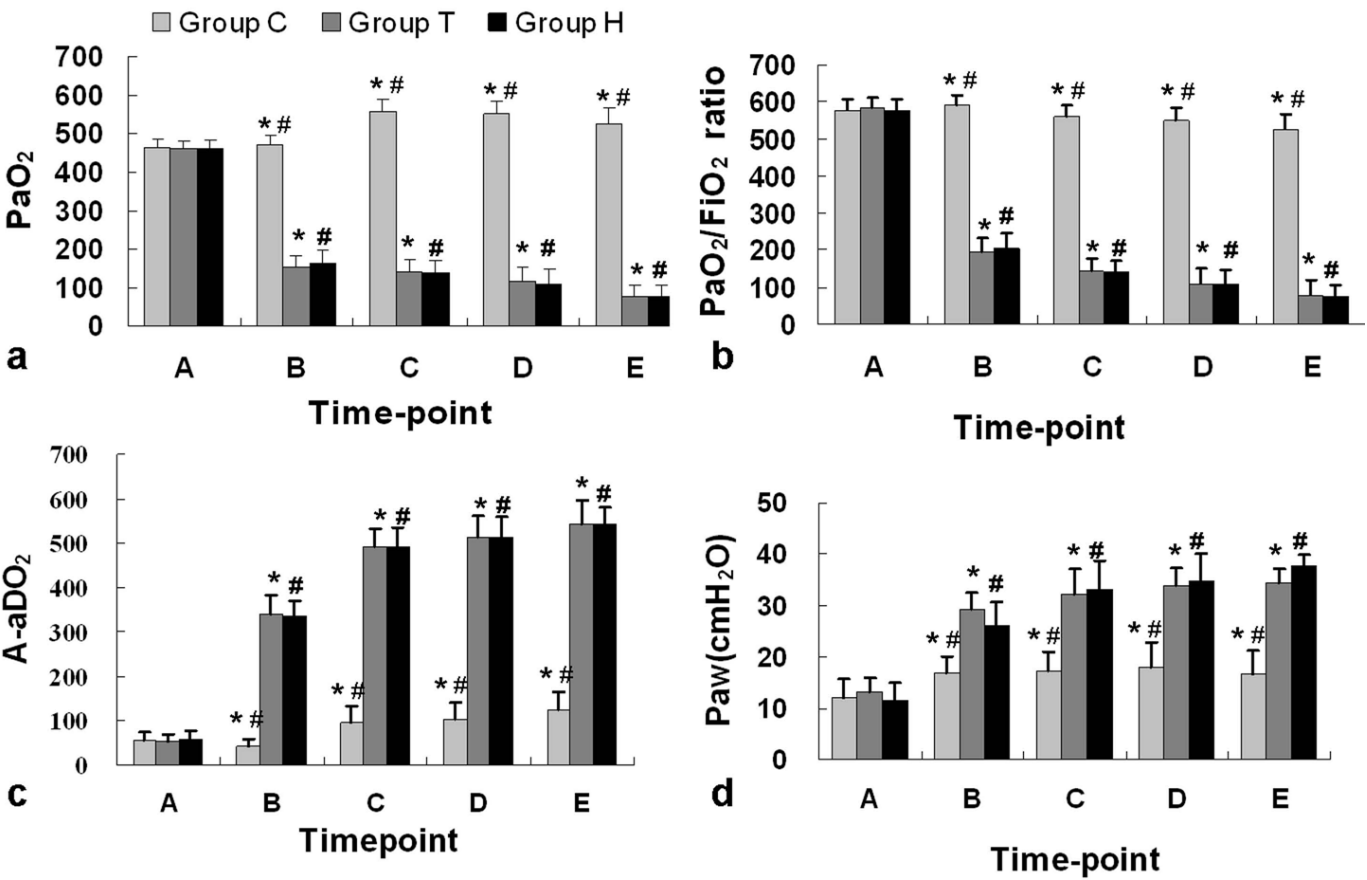
**The change of pulmonary function**. a, the change of PaO_2_; b, the change of P/F; c, the change of A-aDO_2_; d, the change of Paw. *, *p *< 0.05, group T comparing with group C; #, *p *< 0.05 group H comparing with group C.

Respiratory and blood gas test data were shown in Figure 3. The respiratory and blood gas data had no significant difference between groups at the baseline. After OA administration, the PaO_2 _significantly dropt from 464.4 ± 25.5 mmHg of time-point A to 154.9 ± 29.8 mmHg of time-point B in group T (*p *< 0.001) and from 460.9 ± 24.2 mmHg to 164.7 ± 33.0 mmHg in group H (*p *< 0.001), while that of group C had no significant change (*p *= 0.921). The PaO_2 _of group H did not significantly differ from that of group T at every time-points (p > 0.05 respectively). The P/F of group T decreased to 193.6 ± 37.2 mmHg at the time-point B, and achieved the defined standard. Although the P/F of group H at the time-point B was 205.9 ± 41.3 mmHg, which was slightly higher than the standard, it decreased lower than the standard shortly after time-point B and showed a stably decreased trend. A-aDO_2 _in group T and group H significantly increased after OA injury, and showed a gradually elevated trend. P/F and A-aDO_2 _of group C also show slight declined and elevated trend respectively, and were significantly different from those of group H and group T at every time-points (*p *< 0.001 respectively). The Paw of group H and group T sharply increased after OA injection and significantly differed from that of group C at every time-points (p < 0.05 respectively). The arterial PH of group H and group T significantly differed from that of group C at every time-points (p < 0.05 respectively).

### Histopathological assessment

**Figure 4:**
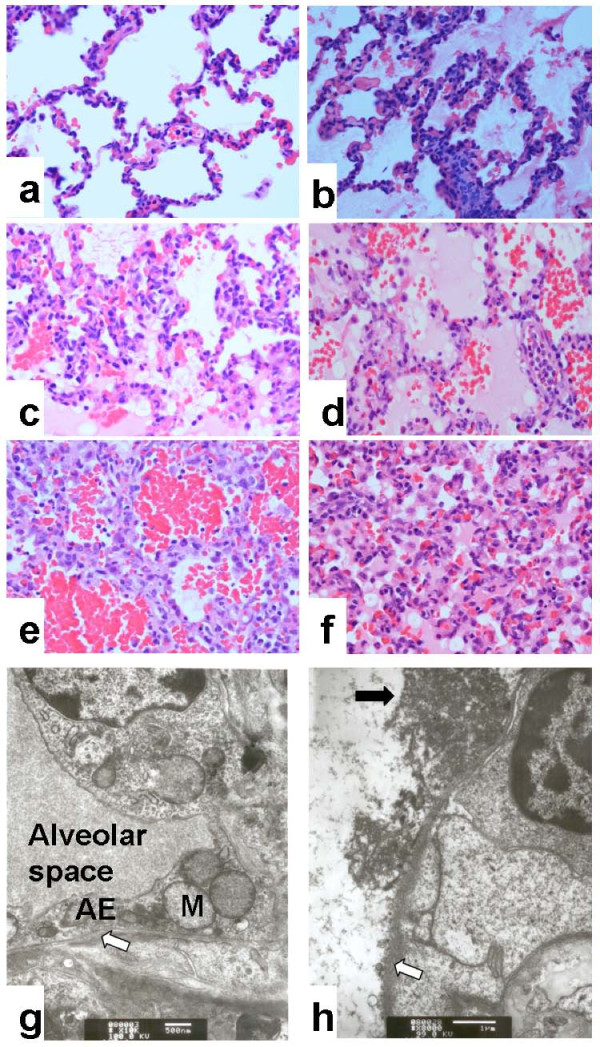
**The typical histopathologic changes of acute lung injury**. a, no hyaline membrane formation; b, mild hyaline membrane formed; c, moderate hyaline membrane appeared; d, severe hyaline membrane formed; e, a great number of erythrocytes appeared in the alveolar cavities with alveolar septa thickening; f, severe hyaline formation with alveolar epitheliums swelling (a, b, c, d, e and f were stained with hematoxylin-eosin stain, ×400); g, disappearance of basement membrane (white arrow) and mitochondria swelling of alveolar epithelium (electron microscopy, ×10,000); h, disappearance of basement membrane (white arrow) and hyaline membrane formation (black arrow)(electron microscopy, ×8,000); AE, alveolar epithelium; M, mitochondria.

Figure 4 showed the histopathologic changes in the three groups. Slides of group H and group T showed typical ALI histopathologic changes under light microscope. The quantification indicated that both of group H and group T had more severe injury than group C (*p *< 0.001 respectively), and no significant difference was found between group T and group H (4.5 ± 1.3 vs. 5.3 ± 0.8, *p *= 0.208). Electric microscopy showed varied degrees of injury to alveolar epithelium and basement membrane.

W/D of group H and group T were almost two-fold more than that of group C (10.77 ± 1.53 vs. 4.84 ± 0.87, *p *< 0.001; 9.73 ± 2.10 vs. 4.84 ± 0.87, *p *< 0.001).

## Discussion

In the present study, the authors established a novel hemodynamically stable two-hit OA-induced ALI animal model in piglets whose body weights and corresponding ages are comparable with those of pediatric ALI patients.

The ALI in pediatric patients often results in high mortality and financial costs [16,17], so it is essential to establish ALI animal model whose body weight and corresponding age are comparable with pediatric patients'. Unfortunately, to our knowledge, most of the OA-induced ALI animal models reported previously were established in adult animals [18-21]. This may be for the concerns of hemodynamic stability. Piglets are more susceptible to injury and more likely to produce hemodynamic fluctuation because of immaturity of their organs, so few experiments adopt piglets as subjects. In the present study, the authors intended to establish a hemodynamically stable OA-induced ALI animal model in piglets whose corresponding age and body weight were comparable with pediatric patients', and thereby piglets were chosen.

There are several papers reporting OA-induced ALI animals in which OA is administrated by bolus injection. Unfortunately, this traditional OA administration strategy has a high mortality due to hemodynamic instability [7]. Referring to the concept of "two-hit", we established a hemodynamically stable ALI model by infusing oleic acid in two stages. In the first stage, two-thirds of total dosage was injected slowly serving as the fist hit. The other one-third was administrated 1 hr after the first stage, serving as the second hit. Traditional administration patterns of OA reported previously always result in the sharp declines of Psa and CO [11,22]. It was previously reported that almost all of the OA-induced hypotension occurred within 0.5–1 hrs after OA administration accompanied by an increase in PAP and a decrease in CO [18]. In the present study, we also found the sharp decline of Psa and CO accompanied by increase in CVP and PAP in traditionally-OA-administrated group. Two animals died of extremely low Psa in the group 5 to 15 minutes after OA administration. These indicated that traditional infusion patterns might influence the cardiac function. So dopamine is always administrated in the traditional OA administration pattern to maintain hemodynamic stability. In order to record the real hemodynamic change trends and the stability of animal models, dopamine was not used and acidosis was not corrected in the present study. However, the authors found that Pla did not differ significantly among groups, which suggested that OA administration did not influence the left ventricular function. The authors also found that PAP increased shortly after OA administration just as previously reported; and on the other hand, we noticed that the increase of PAP concurred with the decline of CO and Psa. Therefore, we inferred that the increase of PAP caused the decline of CO and Psa, which resulted in the hemodynamic instability. Brimioulle S. and colleagues find that OA can increase both of the pulmonary arterial and venous resistance [23]. But pulmonary hypertension has been shown to predominate in small arteries [24], and pulmonary hypertension in oleic lung injury results not only from functional factors (active vasoconstriction) but also from anatomical factors (microthrombi) [25]. Obviously, bolus injection of OA with the traditional administration pattern might lead to more microthrombi in the pulmonary microcirculation, which might reduce the returning blood volume from pulmonary circulation to left atrium. The decrease of returning blood leads to the drastic increase of PAP and sharp decrease of CO, which resulted in the extremely low Psa. However, in the two-hit animal model, stable and moderate decrease trends of Psa and CO were observed. In the first stage of two-hits, the dosage was just two thirds of the total dosage, which might produce less microthrombi than bolus injection. Therefore, the changes of MPAP, CO and Psa were more moderate. The second hit strengthened the pulmonary injury, but the dosage was only one third of the total dosage, which was not adequate to embolize as many microvessels as the first hit and the traditional OA injection do. In the traditional OA-administrated group, the PAP of the last hr showed a decline trend, while the same phenomenon was not observed in the two-hit group. Obviously, the second hit was essential to maintain the continuous pulmonary injury.

Our data showed that PAP of group T had an instable trend and increased more drastically than that of group H. The Psa of group T decreased more drastically than two-hit group especially shortly after OA administration. The two deaths of group T happened in the Psa-drastically-decreased period. The CVP in these three groups decreased in the late period of protocol, which might contribute to the decrease of Psa at the end of the procedure. All these evidence suggested that the traditional OA administration caused fluctuation of Psa, which possibly serves as the underlying reason of deaths of two animals. However, the first hit in the two-hit group produced moderate lung injury and resulted in moderate hemodynamic changes without sharp fluctuation. The second hit of group H leaded to the further damage to the pulmonary vessels, which produced continuous increase in PAP without sharp decrease of Psa. As for the hemodynamic changes, the two-hit animal model in the present study was more stable than traditional OA-induced animal model.

As for the pulmonary function, both the two-hit pattern and the traditional administration manner produced the same severity of pulmonary injury and gas exchange disorder, which totally matched the defined ALI standard.

## Conclusion

The hypotension in OA-induced ALI animal model may be the result of pulmonary microthrombi and the drastic increase of pulmonary artery pressure. The two-hit ALI animal model induced by OA fulfils the criteria of ALI and has the following characteristics: hemodynamic stability, stable damage to gas exchange and comparability with pediatric patients in body weight and corresponding age. The two-hit ALI animal model can be used to study the basic mechanism and the therapeutic strategies for pediatric ALI patients.

## Competing interests

The authors declare that they have no competing interests.

## Authors' contributions

XFL carried out most of the experiment, the interpretation of the results and the manuscript preparation. YLL, QW and YBZ contributed to the evaluation of the results. JPL carried out the blood gas test. JPL, YLL and QW critically reviewed the manuscript. All authors read and approved the final manuscript.
